# Letter to the Editor: Medicolegal Sidebar: Informed Consent in the Information Age

**DOI:** 10.1007/s11999-015-4665-3

**Published:** 2015-12-16

**Authors:** Anna Zagaja, Rafał K. Patryn

**Affiliations:** Department of Ethics and Human Philosophy, Medical University of Lublin, S. Staszica 4/6, 20-059 Lublin, Poland; Provincial Committee for Adjudication on Medical Events, Czechowska 15, 20-072 Lublin, Poland

*To the Editor,*

We read with interest the Medicolegal Sidebar column on obtaining informed consent in the information age. We agree with the legal doctrine of informed consent, where a doctor must provide enough information to help the patient make enlightened healthcare choices.

Yet physicians frequently omit certain steps in obtaining informed consent. For instance, they often provide insufficient information on harms and risks, do not check if patients understand the provided information, or even ask the nursing staff to obtain the consent for them. Consequently, the patients’ outlook or expectations regarding a treatment method may be distorted.

We have a proposal for improving the process. We would emphasize the importance of how consent is obtained (either on a video, recorded, or in writing). By doing so, we would place more responsibility on the physician and his or her obligation of providing the necessary information and receiving a valid, effective, and informed consent from the patient.

It is the physician’s job to confirm that the patient comprehends what has been presented. But taking into consideration a patient who is going through an emotional breakdown caused by a progressive disease, it is difficult to expect him or her to make treatment choices based on reading intricate and extensive forms, analyzing words and phrases, or asking questions. Here, the question of whether the patient is actually capable of making such a decision comes into play. In many cases, the patient is not informed even though, from a medical perspective, the patient has the capacity to make a decision.

The burden of this process should be placed on the physician, who is legally obliged to provide the patient with information and answer any questions or doubts. Only after everything is clear to the patient does the physician have the right to ask for consent, to which the patient can agree or disagree.

We understand that this proposal requiring a written, audio, or video recording of the information “transaction” between the physician and the patient places an additional burden on the physician. But we feel strongly that by doing so, the physician will be obtaining consent from a more-informed patient.

